# (2,2′-Bipyridine-κ^2^ *N*,*N*′)[bis­(diphenyl­thio­phosphino­yl)meth­yl]lithium(I) benzene monosolvate

**DOI:** 10.1107/S160053681300456X

**Published:** 2013-02-20

**Authors:** Wenshan Ren, Suchada Chantrapromma, Hoong-Kun Fun

**Affiliations:** a College of Chemistry and Chemical Engineering, Southwest University, Chongqing 400715, People’s Republic of China; b Department of Chemistry, Faculty of Science, Prince of Songkla University, Hat-Yai, Songkhla 90112, Thailand; c X-ray Crystallography Unit, School of Physics, Universiti Sains Malaysia, 11800 USM, Penang, Malaysia; d Department of Pharmaceutical Chemistry, College of Pharmacy, King Saud University, Riyadh 11451, Saudi Arabia

## Abstract

In the title benzene-solvated heteroleptic lithium complex, [Li(C_25_H_21_P_2_S_2_)(C_10_H_8_N_2_)]·C_6_H_6_, the Li^I^ ion is four-coordinated in a distorted tetra­hedral geometry by two S atoms and two N atoms of the two chelating ligands, *viz.* bis­(diphenyl­thio­phosphino­yl)methyl and 2,2′-bipyridine. The 2,2′-bipyridine mol­ecule is slightly twisted with a dihedral angle between the pyridine rings of 7.35 (12)°. Intra­molecular C—H⋯S hydrogen bonds are present. In the crystal, mol­ecules are stacked along the *c* axis by π–π inter­actions, with centroid–centroid distances of 3.6021 (15) and 3.6401 (16) Å. The crystal structure also features weak C—H⋯π inter­actions.

## Related literature

For standard bond lengths, see: Allen *et al.* (1987 ▶). For background to and applications of thio­phosphinoyl ligands and their complexes, see: Amir *et al.* (2013 ▶); Leung, Wan & Mak (2010 ▶); Leung, Wan, Kan & Mak (2010 ▶); Ren *et al.* (2011 ▶). For related structures, see: Thirumoorthi & Chivers (2012 ▶).
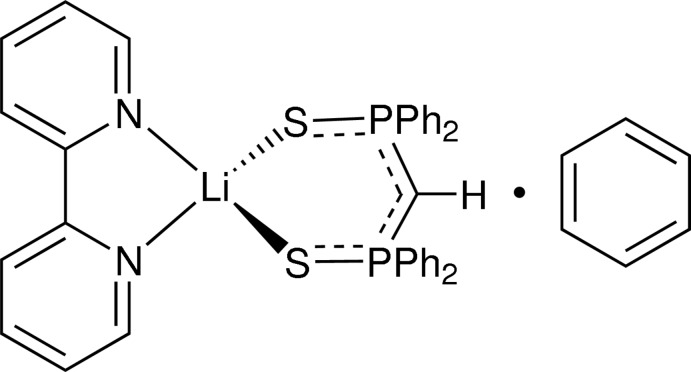


## Experimental

### 

#### Crystal data

[Li(C_25_H_21_P_2_S_2_)(C_10_H_8_N_2_)]·C_6_H_6_
*M* *_r_* = 688.73
Triclinic, 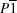

*a* = 10.7654 (11) Å
*b* = 13.1295 (12) Å
*c* = 13.5498 (11) Å
α = 90.746 (2)°
β = 101.902 (1)°
γ = 109.257 (2)°
*V* = 1762.3 (3) Å^3^
*Z* = 2
Mo *K*α radiation
μ = 0.28 mm^−1^
*T* = 297 K
0.44 × 0.28 × 0.20 mm

#### Data collection

Bruker APEXII CCD area detector diffractometer
Absorption correction: multi-scan (*SADABS*; Bruker, 2009 ▶) *T*_min_ = 0.889, *T*_max_ = 0.947
10290 measured reflections
7517 independent reflections
5856 reflections with *I* > 2σ(*I*)
*R*_int_ = 0.031

#### Refinement

*R*[*F* ^2^ > 2σ(*F* ^2^)] = 0.050
*wR*(*F* ^2^) = 0.115
*S* = 1.04
7517 reflections
433 parameters
H-atom parameters constrained
Δρ_max_ = 0.53 e Å^−3^
Δρ_min_ = −0.39 e Å^−3^

### 

Data collection: *APEX2* (Bruker, 2009 ▶); cell refinement: *SAINT* (Bruker, 2009 ▶); data reduction: *SAINT*; program(s) used to solve structure: *SHELXTL* (Sheldrick, 2008 ▶); program(s) used to refine structure: *SHELXTL*; molecular graphics: *SHELXTL*; software used to prepare material for publication: *SHELXTL*, *PLATON* (Spek, 2009 ▶), *Mercury* (Macrae *et al.*, 2006 ▶) and *publCIF* (Westrip, 2010 ▶).

## Supplementary Material

Crystal structure: contains datablock(s) global, I. DOI: 10.1107/S160053681300456X/rz5044sup1.cif

Structure factors: contains datablock(s) I. DOI: 10.1107/S160053681300456X/rz5044Isup2.hkl

Additional supplementary materials:  crystallographic information; 3D view; checkCIF report

## Figures and Tables

**Table 1:** Hydrogen-bond geometry (Å, °) *Cg*3, *Cg*6 and *Cg*7 are the centroids of the C11–C16, C30–C35 and C36–C41 rings, respectively.

*D*—H⋯*A*	*D*—H	H⋯*A*	*D*⋯*A*	*D*—H⋯*A*
C12—H12⋯S1	0.95	2.86	3.367 (2)	114
C25—H25⋯S2	0.95	2.82	3.332 (2)	115
C1—H1⋯*Cg*6	0.95	2.77	3.710 (3)	172
C21—H21⋯*Cg*6^i^	0.95	2.70	3.632 (3)	166
C28—H28⋯*Cg*7^ii^	0.95	2.93	3.654 (3)	134
C37—H37⋯*Cg*3^iii^	0.95	2.80	3.634 (3)	147

Symmetry codes: (i) 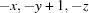; (ii) 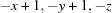; (iii) 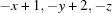.

**Fig. 1.**
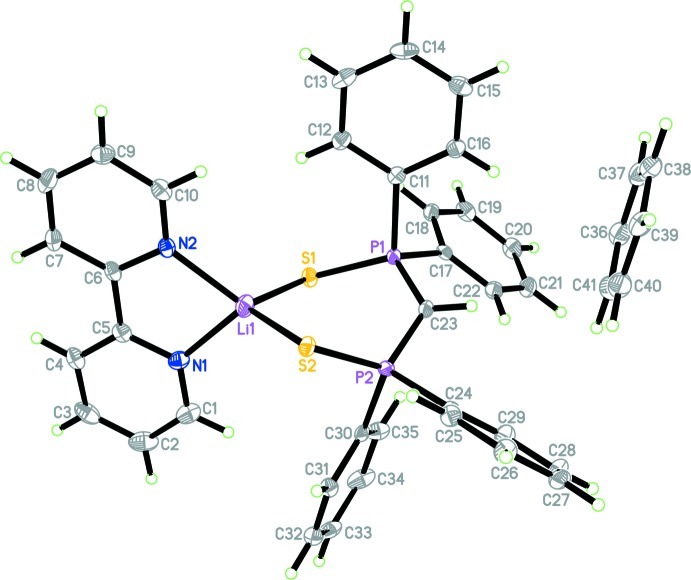
The asymmetric unit of the title compound showing 45% probability displacement ellipsoids.

**Fig. 2.**
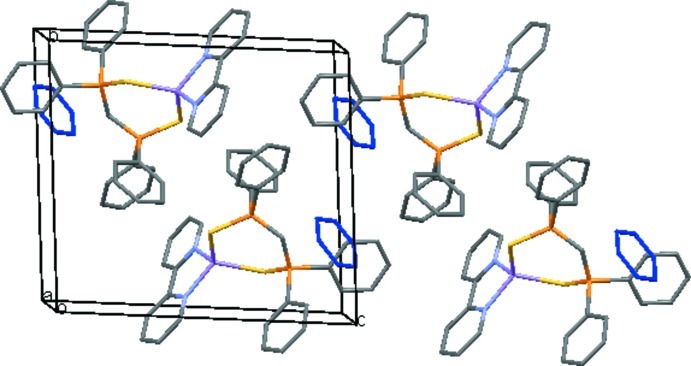
The crystal packing of the title compound viewed along the a axis, showing the arrangement of the molecules. H atoms are omitted for clarity.

**Fig. 3.**
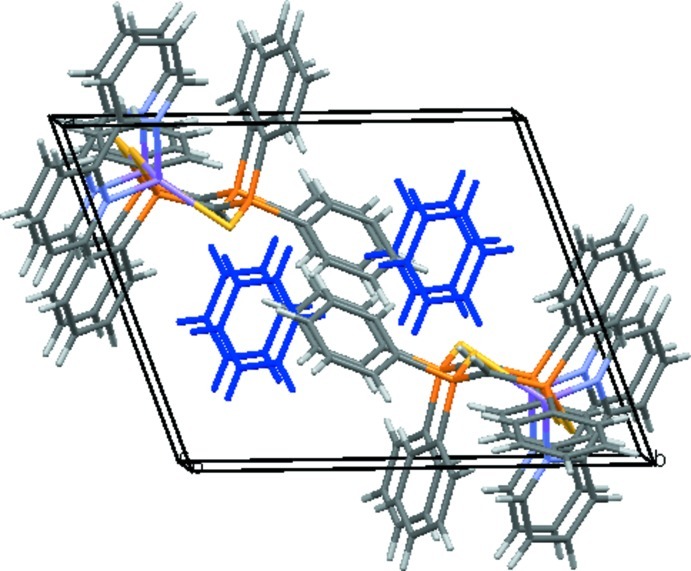
The crystal packing of the title compound showing the molecular stacking along the c axis.

## supplementary crystallographic information

### Comment

Metal chelators containing sulfur are important compounds with many applications such as for metal extraction and development of antioxidant capacity (Amir *et al.*, 2013). Thiophosphinyl ligands and their complexes, including their reactivity and applications, have been extensively studied (Amir *et al.*, 2013; Leung, Wan & Mak, 2010; Leung, Wan, Kan & Mak, 2010; Ren *et al.*, 2011). Our ongoing research on carbene complexes is to synthesize the dilithium salts with the pincer carbene ligand. However the title complex (I) was isolated as a monolithium salt. Herein, the synthesis and crystal strcuture of (I) was reported.

Complex (I) is a heteroleptic lithium(I) complex (Fig. 1) in which the environment around the Li^I^ ion is distorted tetrahedral and the Li^I^ ion is four-coordinated by the two S atoms of bis(diphenylthiophosphinoyl)methyl and two N atoms of 2,2'-bipyridine chelating ligands. The bond angles around the central metal Li^I^ show large deviations from ideal tetrahedral geometry [N1-Li1-S1 = 101.41 (18)°, N1-Li1-S2 = 121.2 (2)°, N2-Li1-S1 = 114.67 (19)°, N2-Li1-S2 = 119.1 (2)°; and the bite angles N1–Li1-N2 = 81.54 (17)° and S1-Li1-S2 = 114.03 (17)°]. The Li-S bond lengths [2.420 (4) and 2.441 (4) Å] and Li-N bond lengths [2.030 (5) and 2.035 (5) Å] are similar to those of the previously reported heteroleptic analogue (Thirumoorthi & Chivers, 2012). Similarly, the P-S [1.9939 (9) and 2.0155 (8) Å] and P-C_methy_ [1.712 (2) and 1.716 (2) Å] bond lengths are comparable to those of another similar Li(I) complex (Thirumoorthi & Chivers, 2012). The six-membered ring Li1–S1–P1–C23–P2–S2 adopts a twisted boat conformation. The 2,2'-bipyridine ring system (C1–C10/N1–N2) is slightly twisted with the dihedral angle between the two pyridine rings being 7.35 (12)°. The dihedral angle between the two C11–C16 and C17–C22 benzene rings is 86.24 (2)° whereas that between the C24–C29 and C30–C35 benzene rings is 85.99 (12)°. The bond lengths of ligand are within normal ranges (Allen *et al.*, 1987). Intramolecular C—H···S hydrogen bonds are observed (Table 1).

The arrangement of molecules in crystal structure of (I) is illustrated in Fig. 2. Fig. 3 shows the stacking of molecules along the *c* axis. The molecules are stacked by π···π interactions with the centroid···centroid distances: *Cg*_1_···*Cg*_2_^iv^ = 3.6012 (15) Å and *Cg*_2_···*Cg*_3_^v^ = 3.6401 (16) Å; *Cg*_1_, *Cg*_2_ and *Cg*_3_ are the centroids of C1–C5/N1, C6–C10/N2 and C11–C16 rings, respectively. C—H···π weak interactions are also present (Table 1).

### Experimental

An n-hexane (2 mL) solution of n-C_4_H_9_Li (0.5 M; 1 mmol) was slowly added into a benzene (10 mL) solution of bis(diphenylthiophosphinoyl)methane (448 mg, 1 mmol) and 2,2'-bipyridine (156 mg, 1 mmol) at 195 K with stirring. After this solution was warmed up to room temperature and stirred for one hour, the solution was filtered. The filtrate was concentrated to about 2 mL under vacuum. Yellow block-shaped single crystals of the title compound suitable for *x*-ray structure determination were obtained when this solution was kept at room temperature for two days. Yield: 585 mg (85%).

### Refinement

Al H atoms were placed in calculated positions with d(C—H) = 0.95 Å and *U*_iso_(H) = 1.2*U*_eq_(C).

### Crystal data

**Table:**

[Li(C_25_H_21_P_2_S_2_)(C_10_H_8_N_2_)]·C_6_H_6_	*Z* = 2
*M**_r_* = 688.73	*F*(000) = 720
Triclinic, *P*1	*D*_x_ = 1.298 Mg m^−^^3^
Hall symbol: -P 1	Mo *K*α radiation, λ = 0.71073 Å
*a* = 10.7654 (11) Å	Cell parameters from 7517 reflections
*b* = 13.1295 (12) Å	θ = 2.2–27.0°
*c* = 13.5498 (11) Å	µ = 0.28 mm^−^^1^
α = 90.746 (2)°	*T* = 297 K
β = 101.902 (1)°	Block, yellow
γ = 109.257 (2)°	0.44 × 0.28 × 0.20 mm
*V* = 1762.3 (3) Å^3^	

### Data collection

**Table:**

Bruker APEXII CCD area detector diffractometer	7517 independent reflections
Radiation source: sealed tube	5856 reflections with *I* > 2σ(*I*)
Graphite monochromator	*R*_int_ = 0.031
φ and ω scans	θ_max_ = 27.0°, θ_min_ = 2.2°
Absorption correction: multi-scan (*SADABS*; Bruker, 2009)	*h* = −11→13
*T*_min_ = 0.889, *T*_max_ = 0.947	*k* = −16→16
10290 measured reflections	*l* = −17→9

### Refinement

**Table:**

Refinement on *F*^2^	Primary atom site location: structure-invariant direct methods
Least-squares matrix: full	Secondary atom site location: difference Fourier map
*R*[*F*^2^ > 2σ(*F*^2^)] = 0.050	Hydrogen site location: inferred from neighbouring sites
*wR*(*F*^2^) = 0.115	H-atom parameters constrained
*S* = 1.04	*w* = 1/[σ^2^(*F*_o_^2^) + (0.035*P*)^2^ + 0.9744*P*] where *P* = (*F*_o_^2^ + 2*F*_c_^2^)/3
7517 reflections	(Δ/σ)_max_ = 0.001
433 parameters	Δρ_max_ = 0.53 e Å^−^^3^
0 restraints	Δρ_min_ = −0.39 e Å^−^^3^

### Special details

**Table:**

Geometry. All esds (except the esd in the dihedral angle between two l.s. planes) are estimated using the full covariance matrix. The cell esds are taken into account individually in the estimation of esds in distances, angles and torsion angles; correlations between esds in cell parameters are only used when they are defined by crystal symmetry. An approximate (isotropic) treatment of cell esds is used for estimating esds involving l.s. planes.
Refinement. Refinement of F^2^ against ALL reflections. The weighted R-factor wR and goodness of fit S are based on F^2^, conventional R-factors R are based on F, with F set to zero for negative F^2^. The threshold expression of F^2^ > 2sigma(F^2^) is used only for calculating R-factors(gt) etc. and is not relevant to the choice of reflections for refinement. R-factors based on F^2^ are statistically about twice as large as those based on F, and R- factors based on ALL data will be even larger.

### Fractional atomic coordinates and isotropic or equivalent isotropic displacement parameters (Å^2^)

**Table:**

	*x*	*y*	*z*	*U*_iso_*/*U*_eq_	
C1	−0.1068 (3)	0.6986 (2)	0.4548 (2)	0.0263 (6)	
H1	−0.1062	0.6450	0.4073	0.032*	
C2	−0.2238 (3)	0.6835 (2)	0.4891 (2)	0.0313 (7)	
H2	−0.3002	0.6196	0.4681	0.038*	
C3	−0.2262 (3)	0.7641 (2)	0.5547 (2)	0.0275 (6)	
H3	−0.3057	0.7571	0.5781	0.033*	
C4	−0.1129 (2)	0.8542 (2)	0.58578 (18)	0.0203 (5)	
H4	−0.1133	0.9105	0.6303	0.024*	
C5	0.0030 (2)	0.86183 (19)	0.55080 (18)	0.0169 (5)	
C6	0.1316 (2)	0.95504 (19)	0.58236 (17)	0.0157 (5)	
C7	0.1516 (3)	1.0354 (2)	0.65794 (18)	0.0200 (5)	
H7	0.0814	1.0335	0.6912	0.024*	
C8	0.2748 (3)	1.1181 (2)	0.6840 (2)	0.0253 (6)	
H8	0.2906	1.1731	0.7359	0.030*	
C9	0.3747 (3)	1.1200 (2)	0.63387 (19)	0.0222 (6)	
H9	0.4600	1.1763	0.6499	0.027*	
C10	0.3469 (2)	1.0375 (2)	0.55958 (19)	0.0199 (5)	
H10	0.4157	1.0388	0.5251	0.024*	
C11	0.3737 (2)	0.95373 (18)	0.19821 (17)	0.0147 (5)	
C12	0.4031 (2)	1.03724 (19)	0.27311 (18)	0.0178 (5)	
H12	0.3388	1.0362	0.3121	0.021*	
C13	0.5256 (3)	1.1220 (2)	0.2912 (2)	0.0230 (6)	
H13	0.5453	1.1781	0.3429	0.028*	
C14	0.6193 (3)	1.1248 (2)	0.2338 (2)	0.0236 (6)	
H14	0.7034	1.1824	0.2467	0.028*	
C15	0.5903 (2)	1.0436 (2)	0.1577 (2)	0.0220 (5)	
H15	0.6538	1.0461	0.1176	0.026*	
C16	0.4683 (2)	0.95842 (19)	0.14008 (18)	0.0184 (5)	
H16	0.4489	0.9027	0.0879	0.022*	
C17	0.1507 (2)	0.82567 (18)	0.04508 (17)	0.0139 (5)	
C18	0.1161 (2)	0.91241 (19)	0.00406 (18)	0.0172 (5)	
H18	0.1333	0.9761	0.0463	0.021*	
C19	0.0566 (2)	0.9061 (2)	−0.09820 (19)	0.0220 (6)	
H19	0.0337	0.9653	−0.1259	0.026*	
C20	0.0309 (3)	0.8129 (2)	−0.15950 (19)	0.0230 (6)	
H20	−0.0115	0.8079	−0.2291	0.028*	
C21	0.0664 (3)	0.7273 (2)	−0.12038 (19)	0.0227 (6)	
H21	0.0506	0.6643	−0.1632	0.027*	
C22	0.1252 (2)	0.73354 (19)	−0.01822 (18)	0.0181 (5)	
H22	0.1484	0.6741	0.0088	0.022*	
C23	0.2662 (2)	0.72073 (18)	0.20470 (17)	0.0148 (5)	
H23	0.3089	0.6989	0.1577	0.018*	
C24	0.3158 (2)	0.53993 (18)	0.28467 (17)	0.0140 (5)	
C25	0.4196 (2)	0.52524 (19)	0.35627 (18)	0.0181 (5)	
H25	0.4558	0.5710	0.4176	0.022*	
C26	0.4708 (2)	0.4439 (2)	0.33868 (19)	0.0215 (5)	
H26	0.5420	0.4343	0.3879	0.026*	
C27	0.4178 (2)	0.37688 (19)	0.2493 (2)	0.0210 (5)	
H27	0.4523	0.3210	0.2375	0.025*	
C28	0.3151 (3)	0.3913 (2)	0.1775 (2)	0.0233 (6)	
H28	0.2790	0.3455	0.1162	0.028*	
C29	0.2647 (2)	0.4725 (2)	0.19484 (19)	0.0207 (5)	
H29	0.1944	0.4823	0.1450	0.025*	
C30	0.0649 (2)	0.56099 (18)	0.29200 (18)	0.0141 (5)	
C31	0.0284 (2)	0.49183 (19)	0.36662 (19)	0.0192 (5)	
H31	0.0952	0.4905	0.4241	0.023*	
C32	−0.1039 (3)	0.4254 (2)	0.3578 (2)	0.0244 (6)	
H32	−0.1274	0.3784	0.4088	0.029*	
C33	−0.2026 (3)	0.4275 (2)	0.2745 (2)	0.0269 (6)	
H33	−0.2937	0.3827	0.2688	0.032*	
C34	−0.1679 (3)	0.4950 (2)	0.1997 (2)	0.0280 (6)	
H34	−0.2355	0.4963	0.1427	0.034*	
C35	−0.0341 (2)	0.5614 (2)	0.2075 (2)	0.0211 (5)	
H35	−0.0106	0.6067	0.1553	0.025*	
C36	0.4914 (3)	0.7279 (2)	−0.0237 (2)	0.0252 (6)	
H36	0.4141	0.7342	−0.0681	0.030*	
C37	0.6179 (3)	0.8037 (2)	−0.0203 (2)	0.0257 (6)	
H37	0.6273	0.8625	−0.0616	0.031*	
C38	0.7308 (3)	0.7930 (2)	0.0440 (2)	0.0261 (6)	
H38	0.8180	0.8440	0.0461	0.031*	
C39	0.7159 (3)	0.7079 (2)	0.1048 (2)	0.0257 (6)	
H39	0.7932	0.7008	0.1486	0.031*	
C40	0.5902 (3)	0.6332 (2)	0.1025 (2)	0.0273 (6)	
H40	0.5805	0.5754	0.1449	0.033*	
C41	0.4776 (3)	0.6435 (2)	0.0373 (2)	0.0271 (6)	
H41	0.3907	0.5920	0.0348	0.032*	
Li1	0.1760 (4)	0.8253 (3)	0.4313 (3)	0.0221 (9)	
N1	0.0051 (2)	0.78456 (16)	0.48517 (16)	0.0190 (4)	
N2	0.22935 (19)	0.95594 (16)	0.53321 (15)	0.0156 (4)	
P1	0.22202 (6)	0.83361 (5)	0.18020 (4)	0.01272 (14)	
P2	0.24198 (6)	0.64311 (5)	0.30422 (4)	0.01215 (14)	
S1	0.09223 (6)	0.85611 (5)	0.25669 (5)	0.01720 (14)	
S2	0.31422 (6)	0.71102 (5)	0.44789 (4)	0.01617 (14)	

### Atomic displacement parameters (Å^2^)

**Table:**

	*U*^11^	*U*^22^	*U*^33^	*U*^12^	*U*^13^	*U*^23^
C1	0.0240 (14)	0.0195 (13)	0.0336 (16)	0.0075 (11)	0.0027 (12)	−0.0003 (11)
C2	0.0170 (13)	0.0250 (15)	0.0457 (18)	0.0021 (12)	0.0012 (12)	0.0063 (13)
C3	0.0168 (13)	0.0353 (16)	0.0339 (16)	0.0103 (12)	0.0102 (12)	0.0147 (13)
C4	0.0200 (13)	0.0285 (14)	0.0175 (13)	0.0137 (11)	0.0064 (10)	0.0080 (10)
C5	0.0190 (12)	0.0187 (12)	0.0165 (12)	0.0111 (10)	0.0034 (10)	0.0072 (10)
C6	0.0173 (12)	0.0209 (12)	0.0128 (12)	0.0106 (10)	0.0048 (9)	0.0060 (9)
C7	0.0239 (13)	0.0239 (13)	0.0166 (13)	0.0123 (11)	0.0072 (10)	0.0021 (10)
C8	0.0338 (15)	0.0230 (14)	0.0183 (13)	0.0112 (12)	0.0015 (11)	−0.0040 (10)
C9	0.0193 (13)	0.0205 (13)	0.0235 (14)	0.0046 (11)	0.0012 (10)	0.0030 (10)
C10	0.0187 (12)	0.0236 (13)	0.0210 (13)	0.0107 (11)	0.0066 (10)	0.0064 (10)
C11	0.0162 (12)	0.0152 (12)	0.0125 (11)	0.0070 (10)	0.0002 (9)	0.0018 (9)
C12	0.0220 (13)	0.0153 (12)	0.0158 (12)	0.0076 (10)	0.0016 (10)	0.0018 (9)
C13	0.0268 (14)	0.0152 (12)	0.0224 (14)	0.0067 (11)	−0.0039 (11)	−0.0011 (10)
C14	0.0163 (12)	0.0172 (13)	0.0295 (15)	0.0007 (10)	−0.0038 (11)	0.0062 (11)
C15	0.0182 (12)	0.0229 (13)	0.0256 (14)	0.0072 (11)	0.0056 (11)	0.0089 (11)
C16	0.0202 (12)	0.0163 (12)	0.0199 (13)	0.0077 (10)	0.0047 (10)	0.0027 (10)
C17	0.0108 (11)	0.0168 (12)	0.0129 (11)	0.0025 (9)	0.0037 (9)	0.0025 (9)
C18	0.0195 (12)	0.0168 (12)	0.0166 (12)	0.0074 (10)	0.0051 (10)	0.0015 (9)
C19	0.0211 (13)	0.0279 (14)	0.0214 (13)	0.0124 (11)	0.0066 (11)	0.0098 (11)
C20	0.0224 (13)	0.0300 (14)	0.0137 (12)	0.0071 (12)	0.0007 (10)	0.0018 (10)
C21	0.0245 (13)	0.0238 (13)	0.0177 (13)	0.0055 (11)	0.0045 (11)	−0.0032 (10)
C22	0.0215 (13)	0.0175 (12)	0.0168 (12)	0.0078 (10)	0.0053 (10)	0.0040 (10)
C23	0.0183 (12)	0.0137 (11)	0.0151 (12)	0.0074 (10)	0.0064 (9)	0.0009 (9)
C24	0.0135 (11)	0.0137 (11)	0.0155 (12)	0.0040 (9)	0.0060 (9)	0.0033 (9)
C25	0.0163 (12)	0.0197 (12)	0.0165 (12)	0.0048 (10)	0.0016 (10)	−0.0005 (10)
C26	0.0196 (13)	0.0251 (14)	0.0239 (14)	0.0131 (11)	0.0042 (10)	0.0051 (11)
C27	0.0220 (13)	0.0150 (12)	0.0316 (15)	0.0103 (11)	0.0110 (11)	0.0052 (10)
C28	0.0247 (14)	0.0194 (13)	0.0250 (14)	0.0084 (11)	0.0026 (11)	−0.0047 (10)
C29	0.0192 (12)	0.0226 (13)	0.0217 (13)	0.0105 (11)	0.0021 (10)	0.0000 (10)
C30	0.0129 (11)	0.0124 (11)	0.0194 (12)	0.0060 (9)	0.0061 (9)	−0.0008 (9)
C31	0.0186 (12)	0.0183 (12)	0.0208 (13)	0.0048 (10)	0.0073 (10)	−0.0003 (10)
C32	0.0258 (14)	0.0177 (13)	0.0292 (15)	0.0023 (11)	0.0139 (12)	−0.0011 (11)
C33	0.0154 (13)	0.0143 (12)	0.0498 (18)	0.0020 (11)	0.0100 (12)	−0.0054 (12)
C34	0.0159 (13)	0.0186 (13)	0.0440 (17)	0.0058 (11)	−0.0050 (12)	−0.0020 (12)
C35	0.0170 (12)	0.0170 (12)	0.0283 (14)	0.0068 (10)	0.0009 (11)	0.0033 (10)
C36	0.0294 (14)	0.0246 (14)	0.0238 (14)	0.0121 (12)	0.0061 (11)	0.0017 (11)
C37	0.0368 (16)	0.0221 (14)	0.0245 (14)	0.0161 (12)	0.0101 (12)	0.0045 (11)
C38	0.0269 (14)	0.0196 (13)	0.0313 (15)	0.0064 (11)	0.0082 (12)	−0.0033 (11)
C39	0.0290 (14)	0.0234 (14)	0.0259 (14)	0.0135 (12)	0.0012 (11)	−0.0013 (11)
C40	0.0378 (16)	0.0251 (14)	0.0231 (14)	0.0145 (13)	0.0091 (12)	0.0051 (11)
C41	0.0280 (15)	0.0282 (15)	0.0259 (15)	0.0083 (12)	0.0102 (12)	0.0002 (11)
Li1	0.023 (2)	0.022 (2)	0.025 (2)	0.0119 (19)	0.0070 (18)	−0.0009 (18)
N1	0.0184 (10)	0.0162 (10)	0.0232 (11)	0.0076 (9)	0.0032 (9)	0.0029 (8)
N2	0.0159 (10)	0.0184 (10)	0.0151 (10)	0.0085 (9)	0.0044 (8)	0.0024 (8)
P1	0.0142 (3)	0.0118 (3)	0.0133 (3)	0.0053 (2)	0.0040 (2)	0.0011 (2)
P2	0.0119 (3)	0.0115 (3)	0.0130 (3)	0.0039 (2)	0.0028 (2)	0.0008 (2)
S1	0.0183 (3)	0.0199 (3)	0.0171 (3)	0.0095 (3)	0.0069 (2)	0.0020 (2)
S2	0.0169 (3)	0.0176 (3)	0.0135 (3)	0.0059 (2)	0.0025 (2)	−0.0013 (2)

### Geometric parameters (Å, º)

**Table:**

C1—N1	1.334 (3)	C23—P2	1.712 (2)
C1—C2	1.386 (4)	C23—P1	1.716 (2)
C1—H1	0.9500	C23—H23	0.9500
C2—C3	1.383 (4)	C24—C25	1.388 (3)
C2—H2	0.9500	C24—C29	1.394 (3)
C3—C4	1.375 (4)	C24—P2	1.824 (2)
C3—H3	0.9500	C25—C26	1.392 (3)
C4—C5	1.398 (3)	C25—H25	0.9500
C4—H4	0.9500	C26—C27	1.386 (3)
C5—N1	1.349 (3)	C26—H26	0.9500
C5—C6	1.490 (3)	C27—C28	1.380 (3)
C6—N2	1.354 (3)	C27—H27	0.9500
C6—C7	1.393 (3)	C28—C29	1.383 (3)
C7—C8	1.382 (4)	C28—H28	0.9500
C7—H7	0.9500	C29—H29	0.9500
C8—C9	1.379 (4)	C30—C35	1.396 (3)
C8—H8	0.9500	C30—C31	1.398 (3)
C9—C10	1.382 (3)	C30—P2	1.825 (2)
C9—H9	0.9500	C31—C32	1.382 (3)
C10—N2	1.336 (3)	C31—H31	0.9500
C10—H10	0.9500	C32—C33	1.389 (4)
C11—C12	1.394 (3)	C32—H32	0.9500
C11—C16	1.397 (3)	C33—C34	1.383 (4)
C11—P1	1.828 (2)	C33—H33	0.9500
C12—C13	1.389 (3)	C34—C35	1.397 (3)
C12—H12	0.9500	C34—H34	0.9500
C13—C14	1.386 (4)	C35—H35	0.9500
C13—H13	0.9500	C36—C41	1.377 (4)
C14—C15	1.385 (4)	C36—C37	1.389 (4)
C14—H14	0.9500	C36—H36	0.9500
C15—C16	1.388 (3)	C37—C38	1.391 (4)
C15—H15	0.9500	C37—H37	0.9500
C16—H16	0.9500	C38—C39	1.383 (4)
C17—C22	1.390 (3)	C38—H38	0.9500
C17—C18	1.398 (3)	C39—C40	1.378 (4)
C17—P1	1.821 (2)	C39—H39	0.9500
C18—C19	1.390 (3)	C40—C41	1.392 (4)
C18—H18	0.9500	C40—H40	0.9500
C19—C20	1.385 (4)	C41—H41	0.9500
C19—H19	0.9500	Li1—N1	2.030 (5)
C20—C21	1.381 (4)	Li1—N2	2.035 (5)
C20—H20	0.9500	Li1—S2	2.420 (4)
C21—C22	1.387 (3)	Li1—S1	2.441 (4)
C21—H21	0.9500	P1—S1	1.9939 (9)
C22—H22	0.9500	P2—S2	2.0155 (8)
			
N1—C1—C2	123.4 (3)	C27—C26—C25	119.9 (2)
N1—C1—H1	118.3	C27—C26—H26	120.0
C2—C1—H1	118.3	C25—C26—H26	120.0
C3—C2—C1	118.1 (3)	C28—C27—C26	120.1 (2)
C3—C2—H2	121.0	C28—C27—H27	120.0
C1—C2—H2	121.0	C26—C27—H27	120.0
C4—C3—C2	119.6 (2)	C27—C28—C29	120.0 (2)
C4—C3—H3	120.2	C27—C28—H28	120.0
C2—C3—H3	120.2	C29—C28—H28	120.0
C3—C4—C5	119.0 (2)	C28—C29—C24	120.8 (2)
C3—C4—H4	120.5	C28—C29—H29	119.6
C5—C4—H4	120.5	C24—C29—H29	119.6
N1—C5—C4	121.7 (2)	C35—C30—C31	119.0 (2)
N1—C5—C6	115.5 (2)	C35—C30—P2	121.66 (18)
C4—C5—C6	122.8 (2)	C31—C30—P2	119.27 (18)
N2—C6—C7	121.8 (2)	C32—C31—C30	120.8 (2)
N2—C6—C5	115.4 (2)	C32—C31—H31	119.6
C7—C6—C5	122.8 (2)	C30—C31—H31	119.6
C8—C7—C6	119.2 (2)	C31—C32—C33	120.0 (2)
C8—C7—H7	120.4	C31—C32—H32	120.0
C6—C7—H7	120.4	C33—C32—H32	120.0
C9—C8—C7	119.4 (2)	C34—C33—C32	119.9 (2)
C9—C8—H8	120.3	C34—C33—H33	120.1
C7—C8—H8	120.3	C32—C33—H33	120.1
C8—C9—C10	118.0 (2)	C33—C34—C35	120.4 (2)
C8—C9—H9	121.0	C33—C34—H34	119.8
C10—C9—H9	121.0	C35—C34—H34	119.8
N2—C10—C9	124.2 (2)	C30—C35—C34	119.9 (2)
N2—C10—H10	117.9	C30—C35—H35	120.1
C9—C10—H10	117.9	C34—C35—H35	120.1
C12—C11—C16	118.7 (2)	C41—C36—C37	120.2 (3)
C12—C11—P1	122.17 (18)	C41—C36—H36	119.9
C16—C11—P1	118.92 (17)	C37—C36—H36	119.9
C13—C12—C11	120.5 (2)	C36—C37—C38	119.5 (2)
C13—C12—H12	119.8	C36—C37—H37	120.2
C11—C12—H12	119.8	C38—C37—H37	120.2
C14—C13—C12	120.1 (2)	C39—C38—C37	119.8 (3)
C14—C13—H13	119.9	C39—C38—H38	120.1
C12—C13—H13	119.9	C37—C38—H38	120.1
C15—C14—C13	120.0 (2)	C40—C39—C38	120.8 (3)
C15—C14—H14	120.0	C40—C39—H39	119.6
C13—C14—H14	120.0	C38—C39—H39	119.6
C14—C15—C16	119.9 (2)	C39—C40—C41	119.2 (3)
C14—C15—H15	120.1	C39—C40—H40	120.4
C16—C15—H15	120.1	C41—C40—H40	120.4
C15—C16—C11	120.7 (2)	C36—C41—C40	120.4 (3)
C15—C16—H16	119.6	C36—C41—H41	119.8
C11—C16—H16	119.6	C40—C41—H41	119.8
C22—C17—C18	118.9 (2)	N1—Li1—N2	81.54 (17)
C22—C17—P1	121.50 (18)	N1—Li1—S2	121.2 (2)
C18—C17—P1	119.61 (18)	N2—Li1—S2	119.1 (2)
C19—C18—C17	120.4 (2)	N1—Li1—S1	101.41 (18)
C19—C18—H18	119.8	N2—Li1—S1	114.67 (19)
C17—C18—H18	119.8	S2—Li1—S1	114.03 (17)
C20—C19—C18	119.6 (2)	C1—N1—C5	118.2 (2)
C20—C19—H19	120.2	C1—N1—Li1	127.7 (2)
C18—C19—H19	120.2	C5—N1—Li1	113.6 (2)
C21—C20—C19	120.6 (2)	C10—N2—C6	117.5 (2)
C21—C20—H20	119.7	C10—N2—Li1	128.9 (2)
C19—C20—H20	119.7	C6—N2—Li1	113.5 (2)
C20—C21—C22	119.7 (2)	C23—P1—C17	108.28 (11)
C20—C21—H21	120.2	C23—P1—C11	109.88 (11)
C22—C21—H21	120.2	C17—P1—C11	102.43 (10)
C21—C22—C17	120.8 (2)	C23—P1—S1	115.53 (9)
C21—C22—H22	119.6	C17—P1—S1	109.04 (8)
C17—C22—H22	119.6	C11—P1—S1	110.84 (8)
P2—C23—P1	127.35 (14)	C23—P2—C24	103.93 (11)
P2—C23—H23	116.3	C23—P2—C30	112.13 (11)
P1—C23—H23	116.3	C24—P2—C30	101.49 (10)
C25—C24—C29	118.9 (2)	C23—P2—S2	120.48 (8)
C25—C24—P2	122.51 (18)	C24—P2—S2	109.57 (8)
C29—C24—P2	118.57 (17)	C30—P2—S2	107.53 (8)
C24—C25—C26	120.4 (2)	P1—S1—Li1	105.29 (10)
C24—C25—H25	119.8	P2—S2—Li1	94.62 (11)
C26—C25—H25	119.8		
			
N1—C1—C2—C3	2.9 (4)	S2—Li1—N1—C1	−63.8 (3)
C1—C2—C3—C4	−1.8 (4)	S1—Li1—N1—C1	63.6 (3)
C2—C3—C4—C5	−0.5 (4)	N2—Li1—N1—C5	5.4 (2)
C3—C4—C5—N1	2.0 (3)	S2—Li1—N1—C5	124.3 (2)
C3—C4—C5—C6	−178.4 (2)	S1—Li1—N1—C5	−108.24 (19)
N1—C5—C6—N2	6.6 (3)	C9—C10—N2—C6	0.4 (3)
C4—C5—C6—N2	−173.0 (2)	C9—C10—N2—Li1	−176.6 (2)
N1—C5—C6—C7	−172.6 (2)	C7—C6—N2—C10	−0.2 (3)
C4—C5—C6—C7	7.8 (3)	C5—C6—N2—C10	−179.4 (2)
N2—C6—C7—C8	−0.4 (4)	C7—C6—N2—Li1	177.3 (2)
C5—C6—C7—C8	178.8 (2)	C5—C6—N2—Li1	−2.0 (3)
C6—C7—C8—C9	0.8 (4)	N1—Li1—N2—C10	175.4 (2)
C7—C8—C9—C10	−0.6 (4)	S2—Li1—N2—C10	54.4 (3)
C8—C9—C10—N2	0.0 (4)	S1—Li1—N2—C10	−85.8 (3)
C16—C11—C12—C13	−1.6 (3)	N1—Li1—N2—C6	−1.7 (2)
P1—C11—C12—C13	173.52 (18)	S2—Li1—N2—C6	−122.8 (2)
C11—C12—C13—C14	0.8 (4)	S1—Li1—N2—C6	97.0 (2)
C12—C13—C14—C15	0.6 (4)	P2—C23—P1—C17	−137.84 (16)
C13—C14—C15—C16	−1.1 (4)	P2—C23—P1—C11	111.03 (17)
C14—C15—C16—C11	0.2 (4)	P2—C23—P1—S1	−15.3 (2)
C12—C11—C16—C15	1.2 (3)	C22—C17—P1—C23	7.2 (2)
P1—C11—C16—C15	−174.16 (18)	C18—C17—P1—C23	−175.07 (18)
C22—C17—C18—C19	0.3 (3)	C22—C17—P1—C11	123.3 (2)
P1—C17—C18—C19	−177.44 (18)	C18—C17—P1—C11	−59.0 (2)
C17—C18—C19—C20	0.4 (4)	C22—C17—P1—S1	−119.22 (18)
C18—C19—C20—C21	−1.4 (4)	C18—C17—P1—S1	58.5 (2)
C19—C20—C21—C22	1.6 (4)	C12—C11—P1—C23	−115.8 (2)
C20—C21—C22—C17	−0.9 (4)	C16—C11—P1—C23	59.4 (2)
C18—C17—C22—C21	0.0 (3)	C12—C11—P1—C17	129.29 (19)
P1—C17—C22—C21	177.68 (19)	C16—C11—P1—C17	−55.5 (2)
C29—C24—C25—C26	−0.4 (4)	C12—C11—P1—S1	13.1 (2)
P2—C24—C25—C26	178.70 (19)	C16—C11—P1—S1	−171.73 (16)
C24—C25—C26—C27	−0.2 (4)	P1—C23—P2—C24	−178.69 (15)
C25—C26—C27—C28	0.5 (4)	P1—C23—P2—C30	72.50 (19)
C26—C27—C28—C29	−0.2 (4)	P1—C23—P2—S2	−55.57 (19)
C27—C28—C29—C24	−0.4 (4)	C25—C24—P2—C23	121.7 (2)
C25—C24—C29—C28	0.7 (4)	C29—C24—P2—C23	−59.1 (2)
P2—C24—C29—C28	−178.4 (2)	C25—C24—P2—C30	−121.7 (2)
C35—C30—C31—C32	−0.6 (3)	C29—C24—P2—C30	57.4 (2)
P2—C30—C31—C32	−177.49 (18)	C25—C24—P2—S2	−8.3 (2)
C30—C31—C32—C33	−0.5 (4)	C29—C24—P2—S2	170.88 (17)
C31—C32—C33—C34	0.9 (4)	C35—C30—P2—C23	2.4 (2)
C32—C33—C34—C35	−0.1 (4)	C31—C30—P2—C23	179.20 (18)
C31—C30—C35—C34	1.4 (3)	C35—C30—P2—C24	−107.9 (2)
P2—C30—C35—C34	178.17 (19)	C31—C30—P2—C24	68.8 (2)
C33—C34—C35—C30	−1.0 (4)	C35—C30—P2—S2	137.06 (18)
C41—C36—C37—C38	0.9 (4)	C31—C30—P2—S2	−46.16 (19)
C36—C37—C38—C39	−0.8 (4)	C23—P1—S1—Li1	45.66 (14)
C37—C38—C39—C40	0.0 (4)	C17—P1—S1—Li1	167.84 (13)
C38—C39—C40—C41	0.6 (4)	C11—P1—S1—Li1	−80.14 (13)
C37—C36—C41—C40	−0.2 (4)	N1—Li1—S1—P1	−156.15 (13)
C39—C40—C41—C36	−0.6 (4)	N2—Li1—S1—P1	118.00 (18)
C2—C1—N1—C5	−1.6 (4)	S2—Li1—S1—P1	−24.21 (19)
C2—C1—N1—Li1	−173.1 (2)	C23—P2—S2—Li1	63.07 (14)
C4—C5—N1—C1	−0.9 (3)	C24—P2—S2—Li1	−176.55 (13)
C6—C5—N1—C1	179.4 (2)	C30—P2—S2—Li1	−67.04 (13)
C4—C5—N1—Li1	171.8 (2)	N1—Li1—S2—P2	100.7 (2)
C6—C5—N1—Li1	−7.9 (3)	N2—Li1—S2—P2	−161.3 (2)
N2—Li1—N1—C1	177.2 (2)	S1—Li1—S2—P2	−20.83 (17)

### Hydrogen-bond geometry (Å, º)

*Cg*3, *Cg*6 and *Cg*7 are the centroids of the C11–C16, C30–C35 and C36–C41 rings, respectively.

**Table:**

*D*—H···*A*	*D*—H	H···*A*	*D*···*A*	*D*—H···*A*
C12—H12···S1	0.95	2.86	3.367 (2)	114
C25—H25···S2	0.95	2.82	3.332 (2)	115
C1—H1···*Cg*6	0.95	2.77	3.710 (3)	172
C21—H21···*Cg*6^i^	0.95	2.70	3.632 (3)	166
C28—H28···*Cg*7^ii^	0.95	2.93	3.654 (3)	134
C37—H37···*Cg*3^iii^	0.95	2.80	3.634 (3)	147

Symmetry codes: (i) −*x*, −*y*+1, −*z*; (ii) −*x*+1, −*y*+1, −*z*; (iii) −*x*+1, −*y*+2, −*z*.
